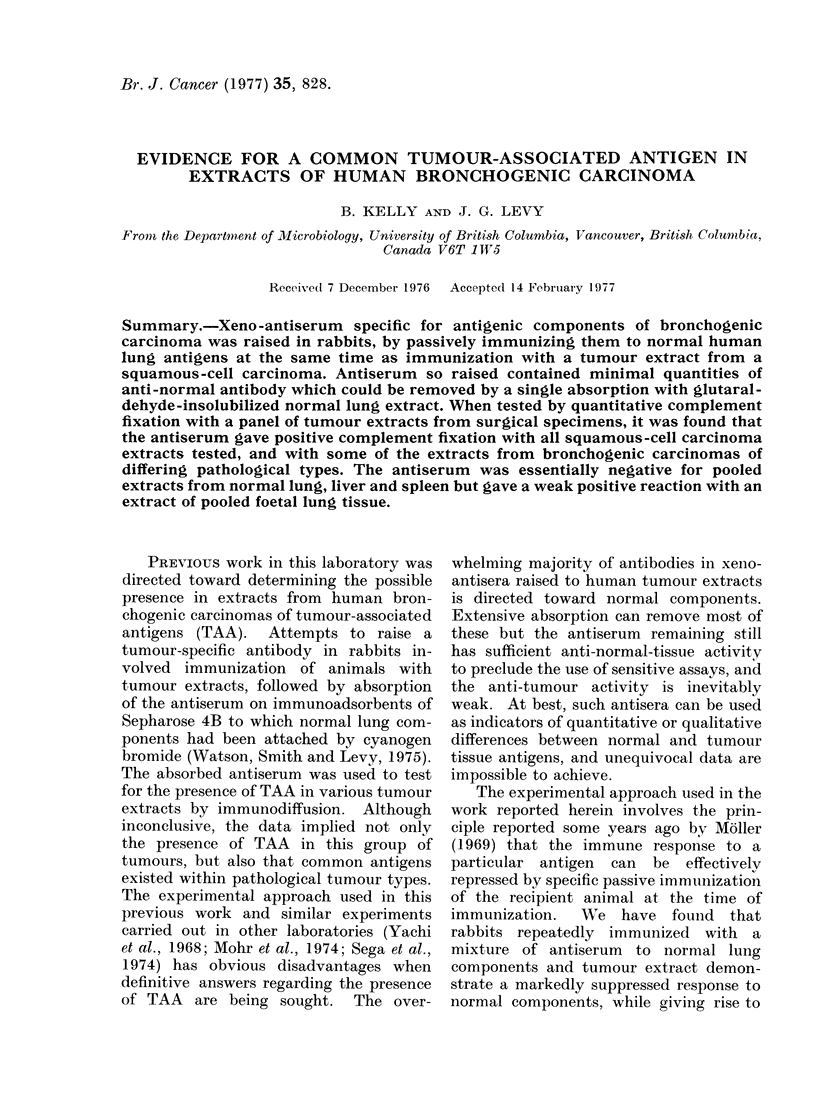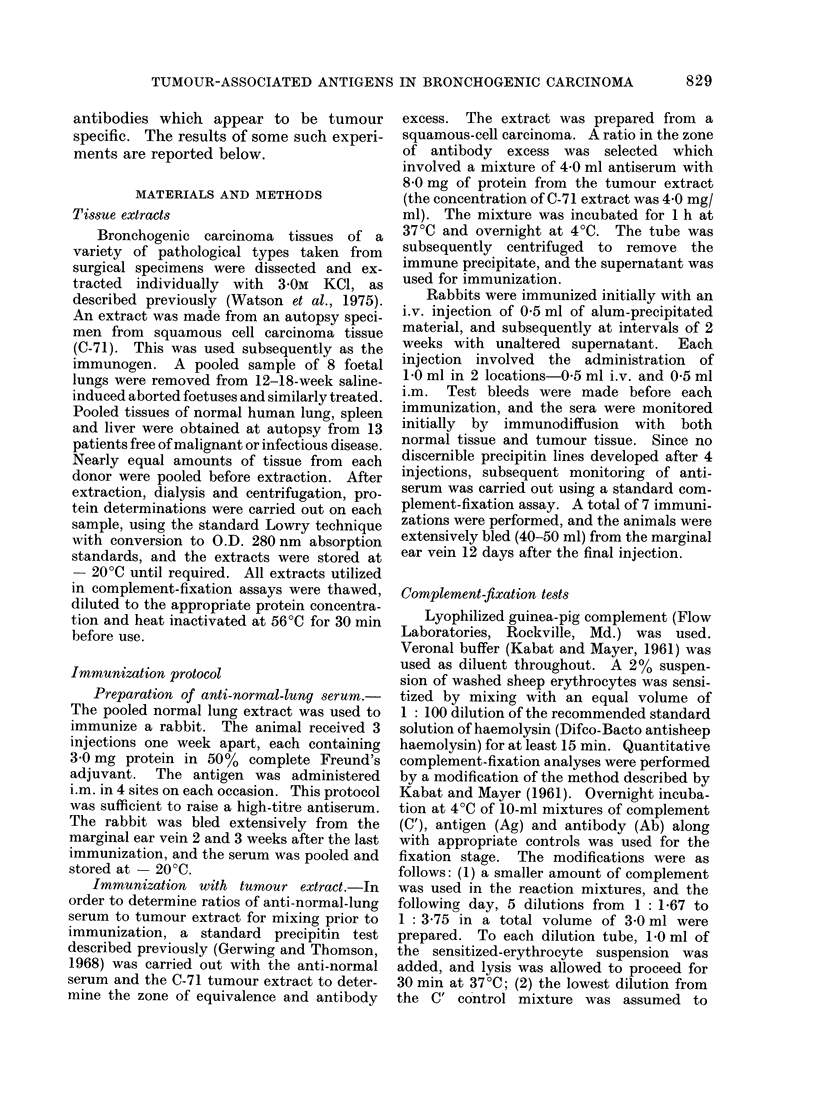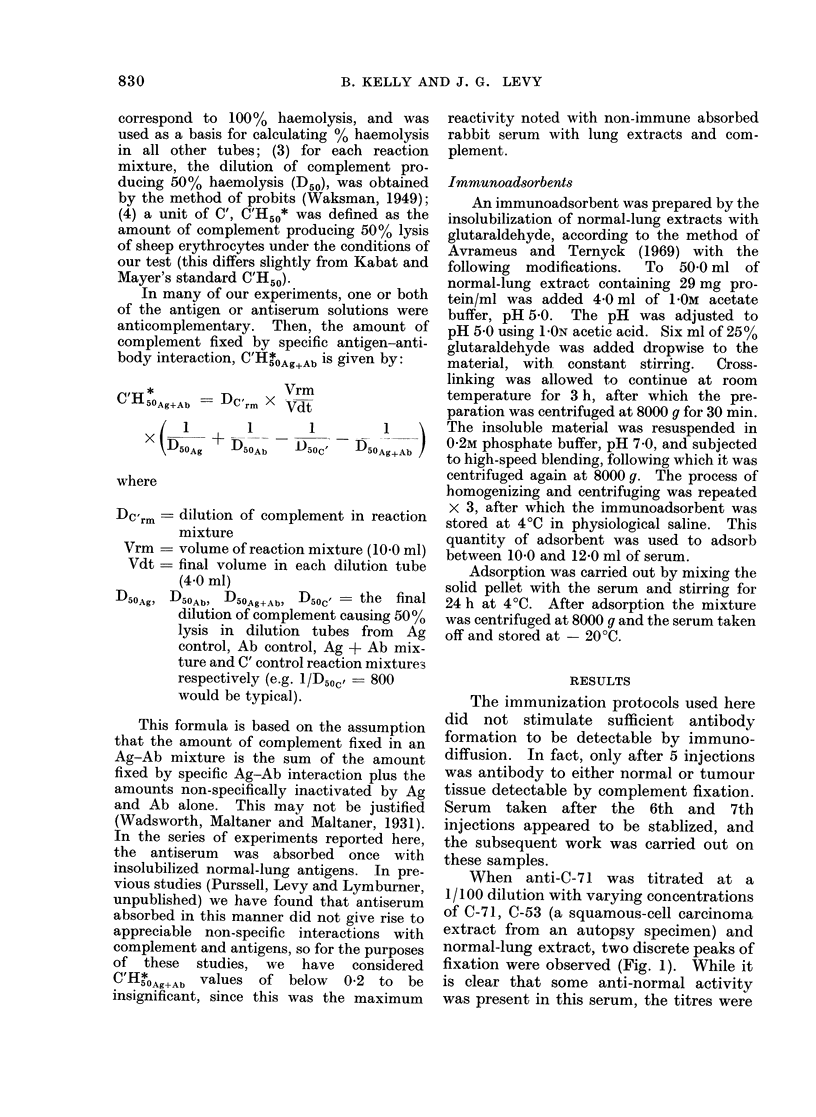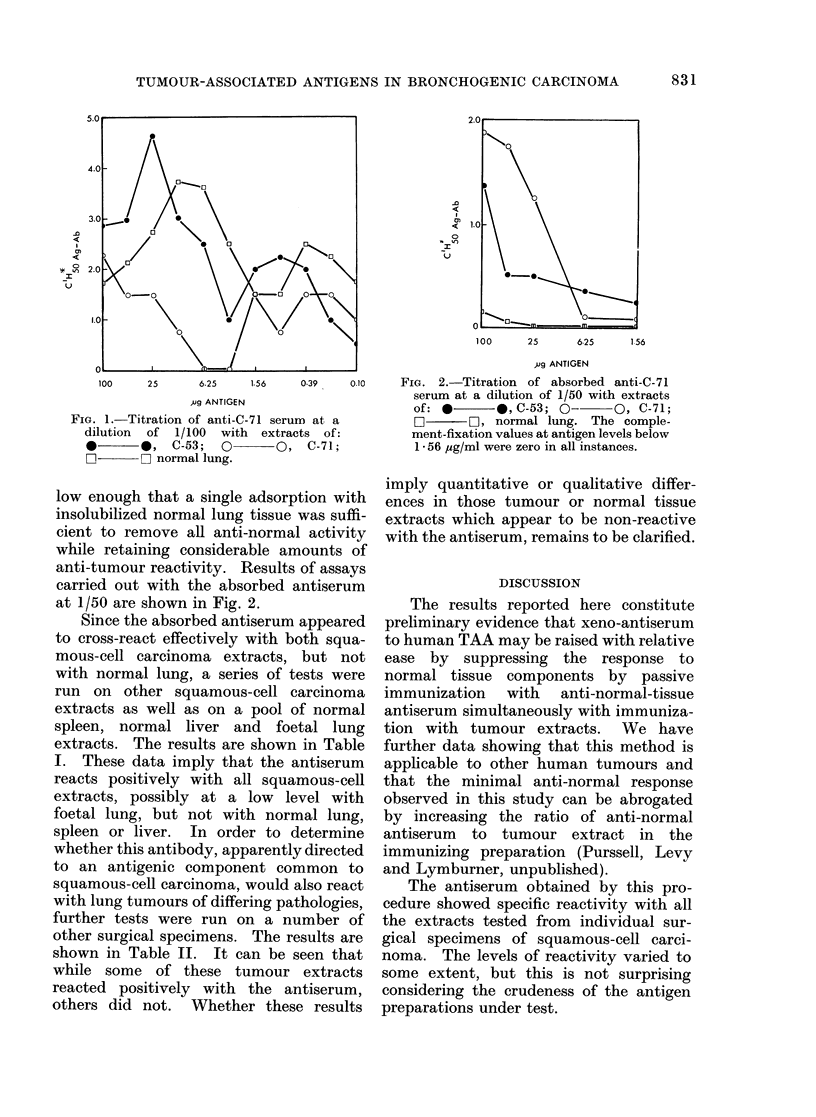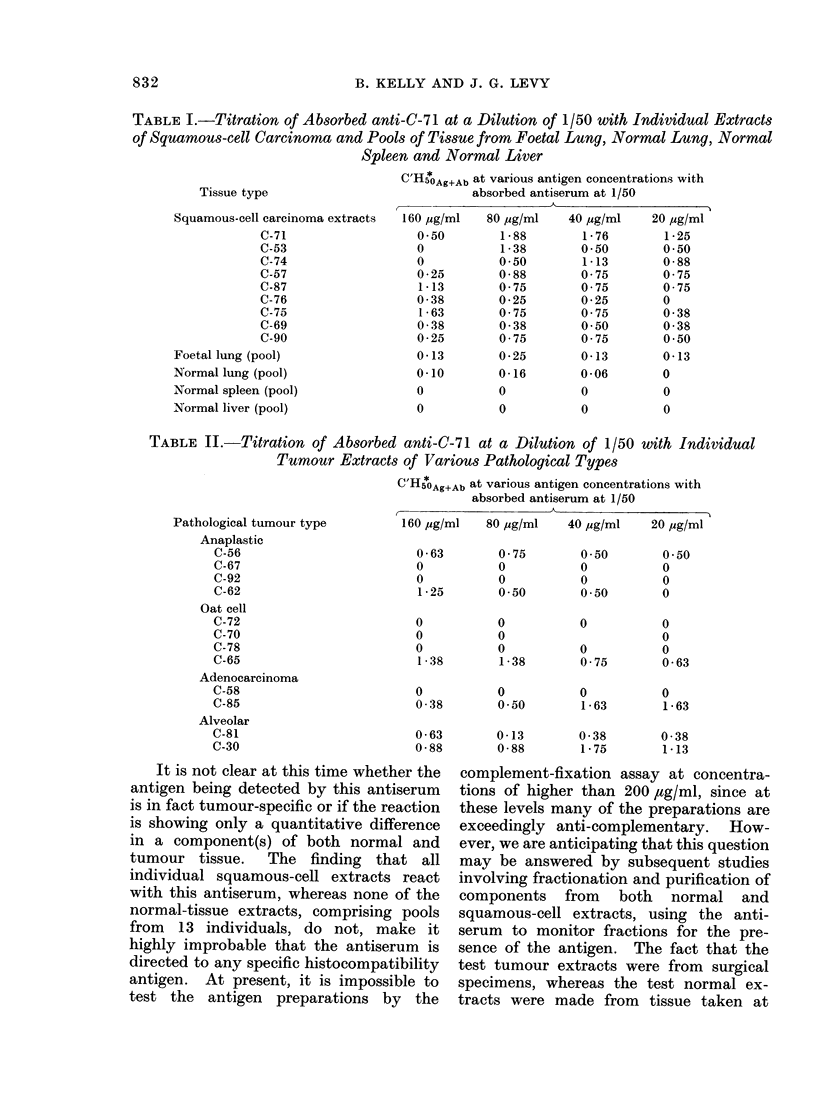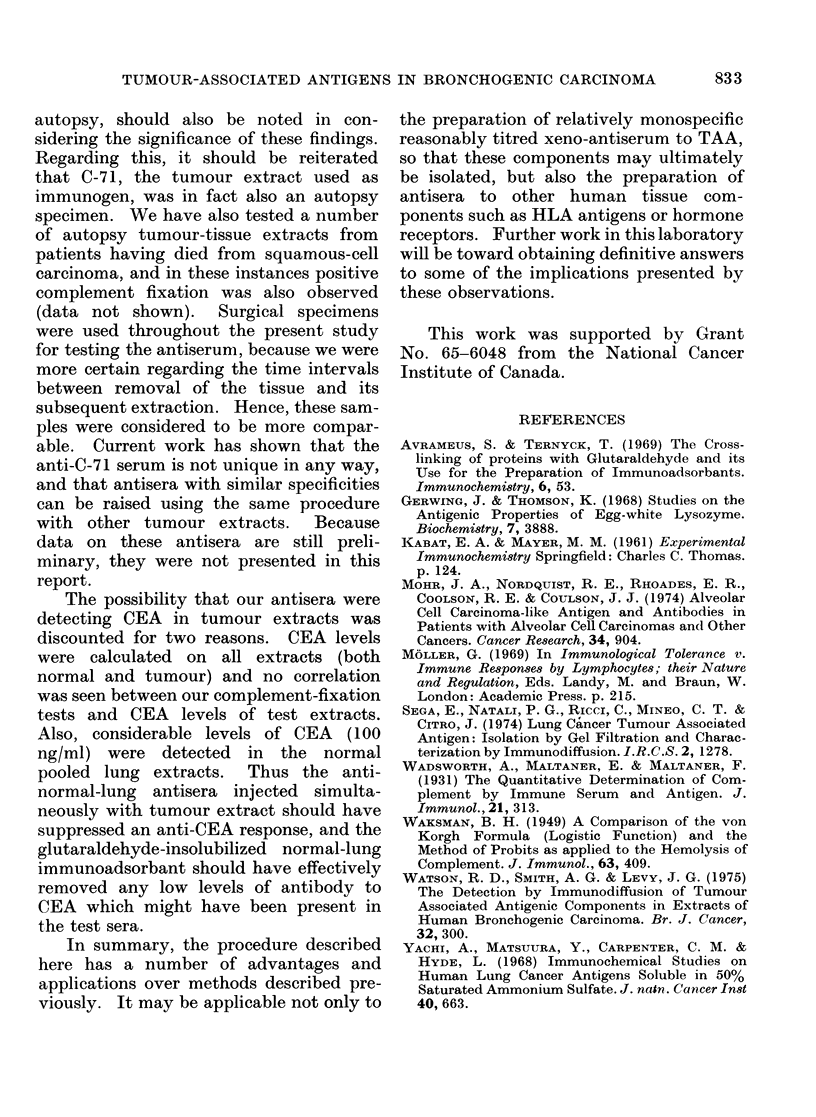# Evidence for a common tumour-associated antigen in extracts of human bronchogenic carcinoma.

**DOI:** 10.1038/bjc.1977.125

**Published:** 1977-06

**Authors:** B. Kelly, J. G. Levy

## Abstract

Xeno-antiserum specific for antigenic components of bronchogenic carcinoma was raised in rabbits, by passively immunizing them to normal human lung antigens at the same time as immunization with a tumour extract from a squamous-cell carcinoma. Antiserum so raised contained minimal quantities of anti-normal antibody which could be removed by a single absorption with glutaral-dehyde-insolubilized normal lung extract. When tested by quantitative complement fixation with a panel of tumour extracts from surgical specimens, it was found that the antiserum gave positive complement fixation with all squamous-cell carcinoma extracts tested, and with some of the extracts from bronchogenic carcinomas of differing pathological types. The antiserum was essentially negative for pooled extracts from normal lung, liver and spleen but gave a weak positive reaction with an extract of pooled foetal lung tissue.


*Br. J. Cancer* (1977) **35**, 828.

### EVIDENCE FOR A COMMON TUMOUR-ASSOCIATED ANTIGEN IN EXTRACTS OF HUMAN BRONCHOGENIC CARCINOMA

B. KELLY AND J. G. LEVY

*From the Department of Microbiology, University of British Columbia, Vancouver, British Columbia, Canada V6T 1W5*

Received 7 December 1976 Accepted 14 February 1977

**Summary.—Xeno-antiserum specific for antigenic components of bronchogenic carcinoma was raised in rabbits, by passively immunizing them to normal human lung antigens at the same time as immunization with a tumour extract from a squamous-cell carcinoma. Antiserum so raised contained minimal quantities of anti-normal antibody which could be removed by a single absorption with glutaraldehyde-insolubilized normal lung extract. When tested by quantitative complement fixation with a panel of tumour extracts from surgical specimens, it was found that the antiserum gave positive complement fixation with all squamous-cell carcinoma extracts tested, and with some of the extracts from bronchogenic carcinomas of differing pathological types. The antiserum was essentially negative for pooled extracts from normal lung, liver and spleen but gave a weak positive reaction with an extract of pooled foetal lung tissue.**

PREVIOUS work in this laboratory was directed toward determining the possible presence in extracts from human bronchogenic carcinomas of tumour-associated antigens (TAA). Attempts to raise a tumour-specific antibody in rabbits involved immunization of animals with tumour extracts, followed by absorption of the antiserum on immunoadsorbents of Sepharose 4B to which normal lung components had been attached by cyanogen bromide (Watson, Smith and Levy, 1975). The absorbed antiserum was used to test for the presence of TAA in various tumour extracts by immunodiffusion. Although inconclusive, the data implied not only the presence of TAA in this group of tumours, but also that common antigens existed within pathological tumour types. The experimental approach used in this previous work and similar experiments carried out in other laboratories (Yachi *et al.*, 1968; Mohr *et al.*, 1974; Sega *et al.*, 1974) has obvious disadvantages when definitive answers regarding the presence of TAA are being sought. The overwhelming majority of antibodies in xeno-antisera raised to human tumour extracts is directed toward normal components. Extensive absorption can remove most of these but the antiserum remaining still has sufficient anti-normal-tissue activity to preclude the use of sensitive assays, and the anti-tumour activity is inevitably weak. At best, such antisera can be used as indicators of quantitative or qualitative differences between normal and tumour tissue antigens, and unequivocal data are impossible to achieve.

The experimental approach used in the work reported herein involves the principle reported some years ago by Möller (1969) that the immune response to a particular antigen can be effectively repressed by specific passive immunization of the recipient animal at the time of immunization. We have found that rabbits repeatedly immunized with a mixture of antiserum to normal lung components and tumour extract demonstrate a markedly suppressed response to normal components, while giving rise to antibodies which appear to be tumour specific. The results of some such experiments are reported below.

### MATERIALS AND METHODS

### *Tissue extracts*

Bronchogenic carcinoma tissues of a variety of pathological types taken from surgical specimens were dissected and extracted individually with 3·0M KCl, as described previously (Watson *et al.*, 1975). An extract was made from an autopsy specimen from squamous cell carcinoma tissue (C-71). This was used subsequently as the immunogen. A pooled sample of 8 foetal lungs were removed from 12–18-week saline-induced aborted foetuses and similarly treated. Pooled tissues of normal human lung, spleen and liver were obtained at autopsy from 13 patients free of malignant or infectious disease. Nearly equal amounts of tissue from each donor were pooled before extraction. After extraction, dialysis and centrifugation, protein determinations were carried out on each sample, using the standard Lowry technique with conversion to O.D. 280 nm absorption standards, and the extracts were stored at − 20°C until required. All extracts utilized in complement-fixation assays were thawed, diluted to the appropriate protein concentration and heat inactivated at 56°C for 30 min before use.

### *Immunization protocol*

*Preparation of anti-normal-lung serum.*—The pooled normal lung extract was used to immunize a rabbit. The animal received 3 injections one week apart, each containing 3·0 mg protein in 50% complete Freund's adjuvant. The antigen was administered i.m. in 4 sites on each occasion. This protocol was sufficient to raise a high-titre antiserum. The rabbit was bled extensively from the marginal ear vein 2 and 3 weeks after the last immunization, and the serum was pooled and stored at − 20°C.

*Immunization with tumour extract.*—In order to determine ratios of anti-normal-lung serum to tumour extract for mixing prior to immunization, a standard precipitin test described previously (Gerwing and Thomson, 1968) was carried out with the anti-normal serum and the C-71 tumour extract to determine the zone of equivalence and antibody excess. The extract was prepared from a squamous-cell carcinoma. A ratio in the zone of antibody excess was selected which involved a mixture of 4·0 ml antiserum with 8·0 mg of protein from the tumour extract (the concentration of C-71 extract was 4·0 mg/ml). The mixture was incubated for 1 h at 37°C and overnight at 4°C. The tube was subsequently centrifuged to remove the immune precipitate, and the supernatant was used for immunization.

Rabbits were immunized initially with an i.v. injection of 0·5 ml of alum-precipitated material, and subsequently at intervals of 2 weeks with unaltered supernatant. Each injection involved the administration of 1·0 ml in 2 locations—0·5 ml i.v. and 0·5 ml i.m. Test bleeds were made before each immunization, and the sera were monitored initially by immunodiffusion with both normal tissue and tumour tissue. Since no discernible precipitin lines developed after 4 injections, subsequent monitoring of antiserum was carried out using a standard complement-fixation assay. A total of 7 immunizations were performed, and the animals were extensively bled (40–50 ml) from the marginal ear vein 12 days after the final injection.

### *Complement-fixation tests*

Lyophilized guinea-pig complement (Flow Laboratories, Rockville, Md.) was used. Veronal buffer (Kabat and Mayer, 1961) was used as diluent throughout. A 2% suspension of washed sheep erythrocytes was sensitized by mixing with an equal volume of 1 : 100 dilution of the recommended standard solution of haemolysin (Difco-Bacto antisheep haemolysin) for at least 15 min. Quantitative complement-fixation analyses were performed by a modification of the method described by Kabat and Mayer (1961). Overnight incubation at 4°C of 10-ml mixtures of complement (C′), antigen (Ag) and antibody (Ab) along with appropriate controls was used for the fixation stage. The modifications were as follows: (1) a smaller amount of complement was used in the reaction mixtures, and the following day, 5 dilutions from 1 : 1·67 to 1 : 3·75 in a total volume of 3·0 ml were prepared. To each dilution tube, 1·0 ml of the sensitized-erythrocyte suspension was added, and lysis was allowed to proceed for 30 min at 37°C; (2) the lowest dilution from the C′ control mixture was assumed to correspond to 100% haemolysis, and was used as a basis for calculating % haemolysis in all other tubes; (3) for each reaction mixture, the dilution of complement producing 50% haemolysis ($D_{50}$), was obtained by the method of probits (Waksman, 1949); (4) a unit of C′, $C'H_{50}^*$ was defined as the amount of complement producing 50% lysis of sheep erythrocytes under the conditions of our test (this differs slightly from Kabat and Mayer's standard $C'H_{50}$).

In many of our experiments, one or both of the antigen or antiserum solutions were anticomplementary. Then, the amount of complement fixed by specific antigen–antibody interaction, $C'H^*_{50_{Ag+Ab}}$ is given by:

$$C'H^*_{50_{Ag+Ab}} = D_{C'_{rm}} \times \frac{Vrm}{Vdt} \times \left(\frac{1}{D_{50_{Ag}}} + \frac{1}{D_{50_{Ab}}} - \frac{1}{D_{50_{C'}}} - \frac{1}{D_{50_{Ag+Ab}}}\right)$$

where

$D_{C'_{rm}}$ = dilution of complement in reaction mixture

Vrm = volume of reaction mixture (10·0 ml)

Vdt = final volume in each dilution tube (4·0 ml)

$D_{50_{Ag}}$, $D_{50_{Ab}}$, $D_{50_{Ag+Ab}}$, $D_{50_{C'}}$ = the final dilution of complement causing 50% lysis in dilution tubes from Ag control, Ab control, Ag + Ab mixture and C′ control reaction mixtures respectively (e.g. $1/D_{50_{C'}} = 800$ would be typical).

This formula is based on the assumption that the amount of complement fixed in an Ag–Ab mixture is the sum of the amount fixed by specific Ag–Ab interaction plus the amounts non-specifically inactivated by Ag and Ab alone. This may not be justified (Wadsworth, Maltaner and Maltaner, 1931). In the series of experiments reported here, the antiserum was absorbed once with insolubilized normal-lung antigens. In previous studies (Purssell, Levy and Lymburner, unpublished) we have found that antiserum absorbed in this manner did not give rise to appreciable non-specific interactions with complement and antigens, so for the purposes of these studies, we have considered $C'H^*_{50_{Ag+Ab}}$ values of below 0·2 to be insignificant, since this was the maximum reactivity noted with non-immune absorbed rabbit serum with lung extracts and complement.

*Immunoadsorbents*

An immunoadsorbent was prepared by the insolubilization of normal-lung extracts with glutaraldehyde, according to the method of Avrameus and Ternyck (1969) with the following modifications. To 50·0 ml of normal-lung extract containing 29 mg protein/ml was added 4·0 ml of 1·0M acetate buffer, pH 5·0. The pH was adjusted to pH 5·0 using 1·0N acetic acid. Six ml of 25% glutaraldehyde was added dropwise to the material, with constant stirring. Cross-linking was allowed to continue at room temperature for 3 h, after which the preparation was centrifuged at 8000 *g* for 30 min. The insoluble material was resuspended in 0·2M phosphate buffer, pH 7·0, and subjected to high-speed blending, following which it was centrifuged again at 8000 *g*. The process of homogenizing and centrifuging was repeated × 3, after which the immunoadsorbent was stored at 4°C in physiological saline. This quantity of adsorbent was used to adsorb between 10·0 and 12·0 ml of serum.

Adsorption was carried out by mixing the solid pellet with the serum and stirring for 24 h at 4°C. After adsorption the mixture was centrifuged at 8000 *g* and the serum taken off and stored at − 20°C.

### RESULTS

The immunization protocols used here did not stimulate sufficient antibody formation to be detectable by immunodiffusion. In fact, only after 5 injections was antibody to either normal or tumour tissue detectable by complement fixation. Serum taken after the 6th and 7th injections appeared to be stablized, and the subsequent work was carried out on these samples.

When anti-C-71 was titrated at a 1/100 dilution with varying concentrations of C-71, C-53 (a squamous-cell carcinoma extract from an autopsy specimen) and normal-lung extract, two discrete peaks of fixation were observed (Fig. 1). While it is clear that some anti-normal activity was present in this serum, the titres were

FIG. 1.—Titration of anti-C-71 serum at a dilution of 1/100 with extracts of: ●——●, C-53; ○——○, C-71; □——□ normal lung.

FIG. 2.—Titration of absorbed anti-C-71 serum at a dilution of 1/50 with extracts of: ●——●, C-53; ○——○, C-71; □——□, normal lung. The complement-fixation values at antigen levels below 1·56 µg/ml were zero in all instances.

low enough that a single adsorption with insolubilized normal lung tissue was sufficient to remove all anti-normal activity while retaining considerable amounts of anti-tumour reactivity. Results of assays carried out with the absorbed antiserum at 1/50 are shown in Fig. 2.

Since the absorbed antiserum appeared to cross-react effectively with both squamous-cell carcinoma extracts, but not with normal lung, a series of tests were run on other squamous-cell carcinoma extracts as well as on a pool of normal spleen, normal liver and foetal lung extracts. The results are shown in Table I. These data imply that the antiserum reacts positively with all squamous-cell extracts, possibly at a low level with foetal lung, but not with normal lung, spleen or liver. In order to determine whether this antibody, apparently directed to an antigenic component common to squamous-cell carcinoma, would also react with lung tumours of differing pathologies, further tests were run on a number of other surgical specimens. The results are shown in Table II. It can be seen that while some of these tumour extracts reacted positively with the antiserum, others did not. Whether these results imply quantitative or qualitative differences in those tumour or normal tissue extracts which appear to be non-reactive with the antiserum, remains to be clarified.

### DISCUSSION

The results reported here constitute preliminary evidence that xeno-antiserum to human TAA may be raised with relative ease by suppressing the response to normal tissue components by passive immunization with anti-normal-tissue antiserum simultaneously with immunization with tumour extracts. We have further data showing that this method is applicable to other human tumours and that the minimal anti-normal response observed in this study can be abrogated by increasing the ratio of anti-normal antiserum to tumour extract in the immunizing preparation (Purssell, Levy and Lymburner, unpublished).

The antiserum obtained by this procedure showed specific reactivity with all the extracts tested from individual surgical specimens of squamous-cell carcinoma. The levels of reactivity varied to some extent, but this is not surprising considering the crudeness of the antigen preparations under test.

TABLE I.—*Titration of Absorbed anti-C-*71 *at a Dilution of* 1/50 *with Individual Extracts of Squamous-cell Carcinoma and Pools of Tissue from Foetal Lung, Normal Lung, Normal Spleen and Normal Liver*

| Tissue type | $C'H^{*}_{50_{Ag+Ab}}$ at various antigen concentrations with absorbed antiserum at 1/50 | | | |
|---|---|---|---|---|
| Squamous-cell carcinoma extracts | 160 µg/ml | 80 µg/ml | 40 µg/ml | 20 µg/ml |
| C-71 | 0·50 | 1·88 | 1·76 | 1·25 |
| C-53 | 0 | 1·38 | 0·50 | 0·50 |
| C-74 | 0 | 0·50 | 1·13 | 0·88 |
| C-57 | 0·25 | 0·88 | 0·75 | 0·75 |
| C-87 | 1·13 | 0·75 | 0·75 | 0·75 |
| C-76 | 0·38 | 0·25 | 0·25 | 0 |
| C-75 | 1·63 | 0·75 | 0·75 | 0·38 |
| C-69 | 0·38 | 0·38 | 0·50 | 0·38 |
| C-90 | 0·25 | 0·75 | 0·75 | 0·50 |
| Foetal lung (pool) | 0·13 | 0·25 | 0·13 | 0·13 |
| Normal lung (pool) | 0·10 | 0·16 | 0·06 | 0 |
| Normal spleen (pool) | 0 | 0 | 0 | 0 |
| Normal liver (pool) | 0 | 0 | 0 | 0 |

TABLE II.—*Titration of Absorbed anti-C-*71 *at a Dilution of* 1/50 *with Individual Tumour Extracts of Various Pathological Types*

| Pathological tumour type | $C'H^{*}_{50_{Ag+Ab}}$ at various antigen concentrations with absorbed antiserum at 1/50 | | | |
|---|---|---|---|---|
| | 160 µg/ml | 80 µg/ml | 40 µg/ml | 20 µg/ml |
| Anaplastic | | | | |
| C-56 | 0·63 | 0·75 | 0·50 | 0·50 |
| C-67 | 0 | 0 | 0 | 0 |
| C-92 | 0 | 0 | 0 | 0 |
| C-62 | 1·25 | 0·50 | 0·50 | 0 |
| Oat cell | | | | |
| C-72 | 0 | 0 | 0 | 0 |
| C-70 | 0 | 0 | | 0 |
| C-78 | 0 | 0 | 0 | 0 |
| C-65 | 1·38 | 1·38 | 0·75 | 0·63 |
| Adenocarcinoma | | | | |
| C-58 | 0 | 0 | 0 | 0 |
| C-85 | 0·38 | 0·50 | 1·63 | 1·63 |
| Alveolar | | | | |
| C-81 | 0·63 | 0·13 | 0·38 | 0·38 |
| C-30 | 0·88 | 0·88 | 1·75 | 1·13 |

It is not clear at this time whether the antigen being detected by this antiserum is in fact tumour-specific or if the reaction is showing only a quantitative difference in a component(s) of both normal and tumour tissue. The finding that all individual squamous-cell extracts react with this antiserum, whereas none of the normal-tissue extracts, comprising pools from 13 individuals, do not, make it highly improbable that the antiserum is directed to any specific histocompatibility antigen. At present, it is impossible to test the antigen preparations by the complement-fixation assay at concentrations of higher than 200 µg/ml, since at these levels many of the preparations are exceedingly anti-complementary. However, we are anticipating that this question may be answered by subsequent studies involving fractionation and purification of components from both normal and squamous-cell extracts, using the antiserum to monitor fractions for the presence of the antigen. The fact that the test tumour extracts were from surgical specimens, whereas the test normal extracts were made from tissue taken at autopsy, should also be noted in considering the significance of these findings. Regarding this, it should be reiterated that C-71, the tumour extract used as immunogen, was in fact also an autopsy specimen. We have also tested a number of autopsy tumour-tissue extracts from patients having died from squamous-cell carcinoma, and in these instances positive complement fixation was also observed (data not shown). Surgical specimens were used throughout the present study for testing the antiserum, because we were more certain regarding the time intervals between removal of the tissue and its subsequent extraction. Hence, these samples were considered to be more comparable. Current work has shown that the anti-C-71 serum is not unique in any way, and that antisera with similar specificities can be raised using the same procedure with other tumour extracts. Because data on these antisera are still preliminary, they were not presented in this report.

The possibility that our antisera were detecting CEA in tumour extracts was discounted for two reasons. CEA levels were calculated on all extracts (both normal and tumour) and no correlation was seen between our complement-fixation tests and CEA levels of test extracts. Also, considerable levels of CEA (100 ng/ml) were detected in the normal pooled lung extracts. Thus the anti-normal-lung antisera injected simultaneously with tumour extract should have suppressed an anti-CEA response, and the glutaraldehyde-insolubilized normal-lung immunoadsorbant should have effectively removed any low levels of antibody to CEA which might have been present in the test sera.

In summary, the procedure described here has a number of advantages and applications over methods described previously. It may be applicable not only to the preparation of relatively monospecific reasonably titred xeno-antiserum to TAA, so that these components may ultimately be isolated, but also the preparation of antisera to other human tissue components such as HLA antigens or hormone receptors. Further work in this laboratory will be toward obtaining definitive answers to some of the implications presented by these observations.

This work was supported by Grant No. 65–6048 from the National Cancer Institute of Canada.

### REFERENCES

AVRAMEUS, S. & TERNYCK, T. (1969) The Cross-linking of proteins with Glutaraldehyde and its Use for the Preparation of Immunoadsorbants. *Immunochemistry*, **6**, 53.

GERWING, J. & THOMSON, K. (1968) Studies on the Antigenic Properties of Egg-white Lysozyme. *Biochemistry*, **7**, 3888.

KABAT, E. A. & MAYER, M. M. (1961) *Experimental Immunochemistry* Springfield: Charles C. Thomas. p. 124.

MOHR, J. A., NORDQUIST, R. E., RHOADES, E. R., COOLSON, R. E. & COULSON, J. J. (1974) Alveolar Cell Carcinoma-like Antigen and Antibodies in Patients with Alveolar Cell Carcinomas and Other Cancers. *Cancer Research*, **34**, 904.

MÖLLER, G. (1969) In *Immunological Tolerance v. Immune Responses by Lymphocytes; their Nature and Regulation*, Eds. Landy, M. and Braun, W. London: Academic Press. p. 215.

SEGA, E., NATALI, P. G., RICCI, C., MINEO, C. T. & CITRO, J. (1974) Lung Cáncer Tumour Associated Antigen: Isolation by Gel Filtration and Characterization by Immunodiffusion. *I.R.C.S.* **2**, 1278.

WADSWORTH, A., MALTANER, E. & MALTANER, F. (1931) The Quantitative Determination of Complement by Immune Serum and Antigen. *J. Immunol.*, **21**, 313.

WAKSMAN, B. H. (1949) A Comparison of the von Korgh Formula (Logistic Function) and the Method of Probits as applied to the Hemolysis of Complement. *J. Immunol.*, **63**, 409.

WATSON, R. D., SMITH, A. G. & LEVY, J. G. (1975) The Detection by Immunodiffusion of Tumour Associated Antigenic Components in Extracts of Human Bronchogenic Carcinoma. *Br. J. Cancer*, **32**, 300.

YACHI, A., MATSUURA, Y., CARPENTER, C. M. & HYDE, L. (1968) Immunochemical Studies on Human Lung Cancer Antigens Soluble in 50% Saturated Ammonium Sulfate. *J. natn. Cancer Inst* **40**, 663.